# Response to Oxidative Burst-Induced Hypoxia Is Associated With Macrophage Inflammatory Profiles as Revealed by Cellular Genome-Wide Association

**DOI:** 10.3389/fimmu.2021.688503

**Published:** 2021-06-18

**Authors:** Mehdi Emam, Saeid Tabatabaei, Mehdi Sargolzaei, Bonnie Mallard

**Affiliations:** ^1^Department of Pathobiology, Ontario Veterinary College, University of Guelph, Guelph, ON, Canada; ^2^Department of Human Genetics, Faculty of Medicine, McGill University, Montreal, QC, Canada; ^3^Select Sires Inc., Plain City, OH, United States; ^4^Center for Genetic Improvement of Livestock, Department of Animal Biosciences, University of Guelph, Guelph, ON, Canada

**Keywords:** inflammation, genetic regulation, hypoixa-inducible factor, macrophage, *E. coli*

## Abstract

**Background:**

In mammalian species, hypoxia is a prominent feature of inflammation. The role of hypoxia in regulating macrophage responses *via* alteration in metabolic pathways is well established. Recently, oxidative burst-induced hypoxia has been shown in murine macrophages after phagocytosis. Despite the available detailed information on the regulation of macrophage function at transcriptomic and epigenomic levels, the association of genetic polymorphism and macrophage function has been less explored. Previously, we have shown that host genetics controls approximately 80% of the variation in an oxidative burst as measured by nitric oxide (NO^-^). Further studies revealed two clusters of transcription factors (hypoxia-related and inflammatory-related) are under the genetic control that shapes macrophages’ pro-inflammatory characteristics.

**Material and Methods:**

In the current study, the association between 43,066 autosomal Single Nucleic Polymorphism (SNPs) and the ability of MDMs in production of NO^-^ in response to *E. coli* was evaluated in 58 Holstein cows. The positional candidate genes near significant SNPs were selected to perform functional analysis. In addition, the interaction between the positional candidate genes and differentially expressed genes from our previous study was investigated.

**Results:**

Sixty SNPs on 22 chromosomes of the bovine genome were found to be significantly associated with NO^-^ production of macrophages. The functional genomic analysis showed a significant interaction between positional candidate genes and mitochondria-related differentially expressed genes from the previous study. Further examination showed 7 SNPs located in the vicinity of genes with roles in response to hypoxia, shaping approximately 73% of the observed individual variation in NO^-^ production by MDM. Regarding the normoxic condition of macrophage culture in this study, it was hypothesized that oxidative burst is responsible for causing hypoxia at the cellular level.

**Conclusion:**

The results suggest that the genetic polymorphism *via* regulation of response to hypoxia is a candidate step that perhaps shapes macrophage functional characteristics in the pathway of phagocytosis leading to oxidative burst, hypoxia, cellular response to hypoxia and finally the pro-inflammatory responses. Since all cells in one individual carry the same alleles, the effect of genetic predisposition of sensitivity to hypoxia will likely be notable on the clinical outcome to a broad range of host-pathogen interactions.

## Introduction

Macrophages are crucial players in both the innate and adaptive immune responses. These cells are equipped with mechanisms to destroy pathogens, regulate inflammation and present antigens to the cells of the adaptive immune system (1, 2). In addition, macrophages are known as effector cells in antibody-mediated immune responses (3). During steady-state, macrophages in some organs (i.e. liver) are generated from embryonic precursor while in some other organs, they are constantly replenished by monocytes (e.g. intestine) (4–6). The difference in the origin of macrophages during infections does not vary between most of the tissues as macrophages are mainly monocyte-derived (5). The genetic and epigenetic mechanisms of the monocyte transformation to macrophages, and biological pathways that are employed by macrophages in various actions, have been reported in detail by various research groups (7–13). However, the genetic polymorphisms that are associated with the magnitude of the macrophage functional characteristics have remained less investigated (14, 15). For instance, the paradigm of macrophage polarization between the two extremes of M1 and M2 in respect to stimuli combination, epigenetic mechanism and the transcriptomic profile have been reported in detail (16), but the effect of host genetic polymorphism on macrophage polarization, epigenetic mechanism and transcriptomic profile have been less investigated. Our group has recently used the magnitude of macrophage nitric oxide (NO^-^) production in response to *Escherichia coli* (*E. coli*) as an index to reveal the effect of host genetic polymorphism on functional characteristics of monocyte-derived macrophages (MDM) (17). This recent study of bovine MDM, as well as another report from a study using a mouse model, have shown that approximately 80% of the individual variation is described by the host genetics (17, 18). The follow up study on the transcriptome of MDM that were classified based on NO^-^ level, showed a distinct inflammatory profile between high and low NO^-^ responder groups. The bioinformatic analysis then revealed that activation of the Hypoxia-induced factor-1α (HIF1A) pathway was positively associated with NO^-^ production ability of MDM (19).

Induction of the HIF1A pathway in macrophages after exposure to pathogens or stimulation with a pathogen-derived component such as lipopolysaccharide has also been reported by other groups (20, 21). From the cellular metabolic point of view, mitochondria are known as the regulators of cellular metabolism and the main intracellular organelle to regulate oxygen and energy metabolites in cellular function (22, 23). The role of mitochondria in response to hypoxia is very well documented (24). In addition, mitochondria have recently gained attention as a master regulator of intracellular signalling with an important role in inflammation (25, 26). Specifically, the role of mitochondria in the regulation of macrophage function and its association with intracellular NAD(P)H content have been reported (27–29). However, the genetic mechanism that might regulate macrophages function *via* mitochondria has remained less clear.

In the current study, it was hypothesized that the magnitude of a macrophage inflammatory response is associated with polymorphism in genes that interact with mitochondria. In order to test this hypothesis, NO^-^ response of bovine MDM against *E. coli* was measured as the phenotypic index of the inflammatory response. A genome-wide association analysis was employed to discover the genetic polymorphisms that are associated with the NO^-^ index. As the last step, the interactions between the genes nearby significant SNPs and mitochondria-related genes were investigated. The results of this study revealed a significant network that was formed between the positional candidate genes and the differentially expressed genes in response to *E. coli* in MDMs that are annotated with “mitochondrion” term.

## Material and Methods

### Animals

Fifty-eight lactating Holstein cows were selected for this study from the University of Guelph Dairy Innovation Centre herd. These animals were not diagnosed nor treated for any diseases in the lactation period when samples were collected. Blood samples were collected as part of a previous study in groups of 12 individuals per sampling visit (17). All the procedure and handling of the animals were approved by the animal care committee of the University of Guelph (AC4449).

### *In Vitro* Transformation of Monocyte to Macrophages

Blood samples were collected from the tail vein in tubes containing EDTA. Blood Mononuclear Cells (BMCs) were purified based on the gradient centrifuge separation method. Concisely as described previously (17), Histopauqe-1077 (Sigma-Aldrich, St. Louis, MO) was loaded into the Sepmate tubes (STEMCELL Technologies, Vancouver, BC) and whole blood samples were overlaid on the top of Histopaque-1077. After centrifugation for 10 minutes at 1200 ×g, the layer of cells above the Histopaque-1077 was collected and washed 3 times with Phosphate Buffered Saline (PBS) to obtain the purified BMCs. Purified BMCs were cultured at the concentration of 1×10^6^ cells per square centimetre of the culture flask (Nunc, Thermo Fisher Scientific Inc., Mississauga, ON) for 2 hours in Monocyte Attachment Medium (PromoCell, Heidelberg, Germany) at 37°C. Non-adherent cells were removed by washing using PBS and the medium was replaced with AIM V^®^ Medium (Thermo Fisher Scientific Inc., Mississauga, ON) containing 5 ng/ml recombinant bovine Granulocyte-Macrophage Colony-Stimulating Factor (GM-CSF, Kingfisher Biotech, St. Paul, MN) in the presence of 5% CO_2_. After 6 days of incubation, adherent cells were detached from the flask using TrypLE™ Select Enzyme (Thermo Fisher Scientific Inc., Mississauga, ON). Phenotypic characteristics of macrophages (CD14^+^, CD205^-^ and strong auto-fluorescence) were analyzed using flow cytometry to determine the proportion of macrophages among the harvested cells (30–32). Harvested cells were labeled with RPE conjugated mouse anti-bovine CD205 (Clone: IL-A114, Bio-Rad, Mississauga, Ontario) and ALEXA FLUOR^®^ 647 conjugated mouse anti-human CD14 (Clone: TÜK4, Bio-Rad, Mississauga, Ontario), separately. The labelled samples were analyzed using the BD Accuri C6 flow cytometer (Becton Dickinson, Franklin Lakes, NJ) and the data from the flow cytometer were analyzed by FlowJo V.10 (FlowJo LLC, Ashland, OR). The fluorescence emission of unlabeled harvested cells was compared with the emission of unlabeled fresh BMCs in 533/30 filter excited by the blue laser to test the auto-fluorescence as described previously (17).

The MDMs from each sample were seeded in 2 wells of a 48-well plate at the density of 2×10^5^ cells per well in AIM V^®^ medium containing 5 ng/ml GM-CSF and incubated overnight. One well was assigned as the control and the other well was assigned as the treatment group and was exposed to inactivated *E. coli* (MOI: 5) for 48 hours. Supernatant from each well was collected and the concentration of nitric oxide (NO^-^) was measured with the Measure-iT™ High-Sensitivity Nitrite Assay Kit (Thermo Fisher Scientific Inc., Mississauga, ON). The concentration of NO^-^ in the challenge group was corrected by the control group. These data collected in the previous study were used in the analyses described below (17).

### Genotyping and Quality Control

DNA was extracted from hair follicles collected from each individual and genotyping was performed with the Illumina Bovine SNP50 BeadChip by Zoetis Canada (Kirkland, Quebec, Canada). The initial dataset contained 45,187 SNP markers that are used in routine official genomic evaluation in Canada by the Lactanet (Guelph, ON, Canada). Details of quality control were explained in Wiggans et al. (33). The sporadic missing genotypes were imputed using 50,000 reference Holsteins from the Lactanet database by FImpute software (34). In the present study SNPs located on the X chromosome were not included and due to relatively small sample size, 1,716 SNPs with minor allele frequency < 1% were excluded resulting in 43,066 SNPs for further association analysis.

### Statistical Analysis

The UNIVARIATE procedure of SAS (V 9.4) was used to test the distribution of the dependent variable (NO^-^ response to *E. coli*) for normal, Log-normal, Weibull, and Gamma distributions. The goodness-of-fit tests based on the empirical distribution function for Gamma distribution were >0.50. The *p values* for goodness-of-fit tests based on the empirical distribution function, Anderson-Darling, Cramer-von Mises, and Kolmogorov-Smirnov, for Gamma distribution, were >0.50, > 0.25 and >0.50, respectively. Therefore, the dependent variable was transformed based on the method described by Krishnamoorthy et al. (2008) to achieve normal distribution (35).

The GLM procedure of SAS (V. 9.4) was used to test the effect of age in month, days in milk (classes: 1 = 0-20 days, 2 = 21-105 days, 3 = 106-235 days, 4 = > 235 days) (36, 37), days in pregnancy (classes: 1 = non pregnant, 2 = 1 - 120 days, 3 = 121 - 180 days, 4 = 181 - 220 days) (37) and sampling group (classes: 1-5). The statistical model was as follow:

$$y_{ijkl}=\mu +\beta_{1}\times a_{i}+g_{j}+m_{k}+p_{l}+e_{ijkl}$$

Where; *y_ijkl_* is the vector of the phenotype [the cubic roots of nitrite concentration of culture supernatant 48 hours after treatment with *E. coli* (n = 58)]; *μ* is the overall average of the response. *β_1_* is the linear coefficient of the fixed regression on age (*a_i_*) (in months), *g_j_* is the fixed effect of *j^th^* class of sampling group, *m_k_* is the fixed effect of *k^th^* class of days in milk, *p_l_* is the fixed effect of *l^th^* class of days in pregnancy and *e_ijkl_* is the random residual effect.

Genome-wide association analysis was carried out by SNP & Variation Suite (ver. 8.8.1, Golden Helix Inc.) using single SNP regression analysis on numerically coded alleles and the cubic roots of NO^-^ concentration as the continuous dependent variable. The statistical model was as follow:

$$y=µ+c\beta +\lambda +e_{i}$$

Where y is the cubic root of nitric oxide concentration in the culture supernatant 48 hours after treatment with *E. coli*, μ is the overall average of the response; *β* is the gene substitution effect for the SNP, λ is the vector of eigenvalues, e is the random residual effects; c is a vector of genotypes for the SNP coded as described, below.

The additive, dominance and recessive models were utilized, separately. For the additive genetic model, the SNP alleles were numerically coded as 0, 1, and 2 for “dd”, “Dd”, and “DD” genotypes, respectively. For the dominance genetic model, alleles were coded as 0 for “dd”; and 1 for “Dd” and “DD”. For the recessive genetic model, alleles were coded as 0 for “dd” and “Dd”; and 1 for “DD”, where d is the major allele and D is the minor allele (38, 39). The principal component analysis was performed on the genotypic data and the top three eigenvalues were included in the analysis to account for the population stratification. The details of the estimators and assumptions are provided in the SNP & Variation Suite (ver. 8.8.1, Golden Helix Inc.) manual. To reduce the probability of false discovery in multiple comparisons, a permutation test was employed by randomizing the observations over the genotypes in 10,000 permutation samples. The permutation value was calculated based on the direct count of the number of samples that have resulted in equal or smaller *p values* compared to the unrandomized *p-value* divided by the total number of samples. The permutation value of 0.0005 was set as the cut-off for the significant association. The linkage between the significant SNPs were evaluated using the Expectation-Maximization algorithm in SNP & Variation Suite (ver. 8.8.1, Golden Helix Inc.) (40). The SNPs with linkage disequilibrium R-Squared index of greater than 0.5 were considered linked and the second SNPs in each pair were removed for the quantitative analysis.

The significant SNPs were used in the K-fold genomic prediction algorithm in SNP & Variation Suite using Bayes C(π) method in 50,000 iterations in 6 folds of prediction (41).

### Bioinformatic Analysis

#### Identifying Positional Candidate Genes

Candidate genes were selected based on two strategies; 1) Position-dependent; and 2) Comparative genomics (42). First, the coordinates of significant SNPs from UMD3.1 were converted to ARS-UCD1.2 by using BovineMine tools from Bovine Genome database. Genes (Ensemble Gene 97) that are located in the vicinity of the significant SNPs (up to 50K bp upstream and downstream of the significant SNPs) were considered as the positional candidate genes. Due to the fact that the annotation of the bovine genome is not complete, the comparative strategy was employed on the SNPs when the first strategy was not able to retrieve any genes. In the comparative strategy, 1Kb up and downstream of the significant SNPs were aligned against the human genome (GRCh38.p12, Ensemble Genes 97), and genes that were located in the identical region were selected as the positional candidate genes. The Gene Ontology (GO) terms for the positional candidate genes were extracted from Ensemble using ‘biomaRt’ (v. 2.41.7) package in R (v. 3.6) (43).

#### Network Analysis

Activation of HIF1A that was found in the previous study, as well as the known role of mitochondria in response to hypoxia led to investigating the interaction between the positional candidate genes and mitochondria, the genes that have been found to be differentially expressed (DE) between the high and low nitric oxide responder were selected from our previous study (GSE136722) (19). Then, GO terms for these genes were extracted from Ensemble using ‘biomaRt’ (v. 2.41.7) package in R (v. 3.6). Only, the DE gene that has been annotated with “mitochondrion” (GO:0005739) were selected for interaction analysis. The protein identifiers of these genes were combined with the list of all positional candidate genes from the current GWAS. The protein-protein interaction network in the final gene set (mitochondria-related DE genes and positional candidate genes from the GWAS) was analyzed using the Search Tool for Retrieval of Interacting Genes/Proteins (STRING, v. 11) (44).

## Results

**Figure 1:**
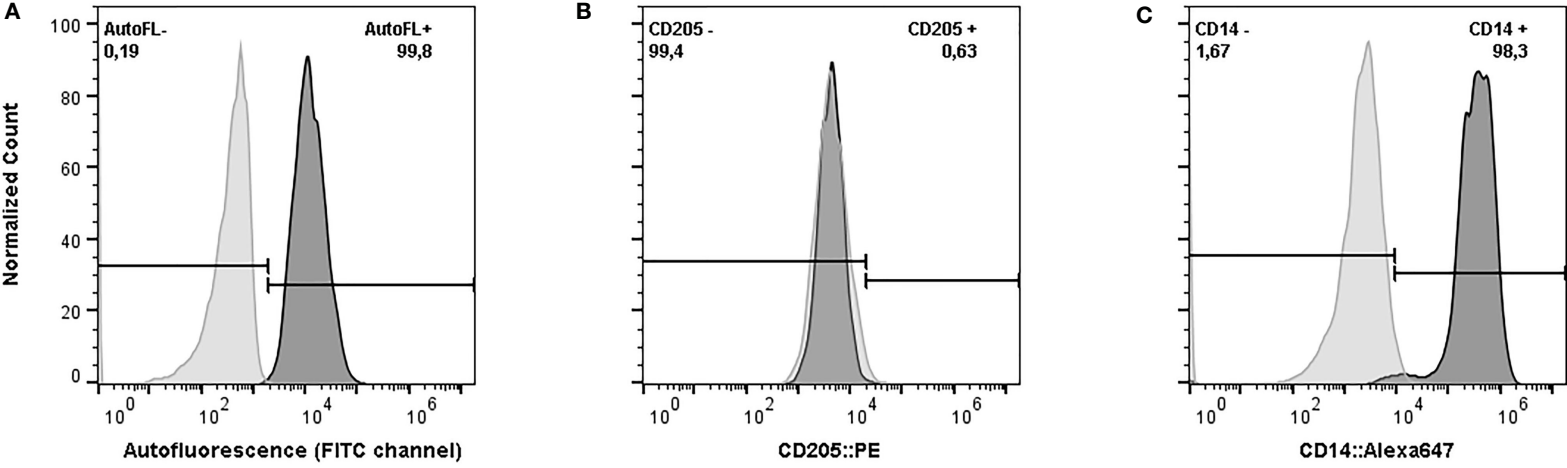
Phenotypic characteristics of harvested cells after six days of *in vitro* incubation in serum-free media supplemented with recombinant bovine granulocyte-macrophage colony-stimulating factor. **(A)** Autofluorescence of Monocyte-Derived Macrophages (MDMs) (dark grey) versus monocyte (light grey), exited by blue laser and the emission was measured by 533/30 filter. **(B)** Expression of CD205 on MDMs. The harvested cells were stained with phytoerythrin (PE) conjugated anti-bovine CD-205 (dark grey) and compared against the unstained sample (light grey). **(C)** Expression of CD14 on MDMs. The harvested cells were stained with Alexa Flour 647 conjugated anti-human CD-14 (Clone TÜK4) (dark grey) versus the unstained sample (light grey). The cells were analyzed in BD Accuri™ C6 cytometer. The data from the flow cytometer were analyzed and graphed using FlowJo (v. 10).

The flow cytometry assay confirmed that more than 98% of the harvested cells after 6 days of incubation had characteristics of macrophages (CD14+, CD205-, and strong autofluorescence in FITC channel) (**Figure 1**). The concentration of NO^-^ in the culture supernatant after 48 hours of exposure to *E. coli* ranged from 0.38 µM up to 18.82 µM (Standard Deviation: 4.07, Coefficient of Variation: 74.78%). The statistical analysis did not show any effect of age (*p-value* = 0.37), group of sampling (*p-value* = 0.25), days in milking (*p-value* = 0.47), or pregnancy (*p-value* = 0.73) on the production of NO^-^ in response to *E. coli* (Model *p-value* = 0.52, R-Squared = 0.21). Therefore, the cubic root of NO^-^ concentrations without any adjustment for environmental effects were used in the association analysis.

**Figure 2:**
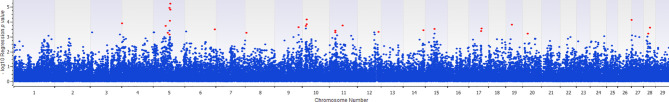
Distribution of -Log10 regression p values from additive genetic model analysis of SNPs in association with MDM nitric oxide production. In this analysis, the alphabetic codes of aa, ab, and bb for each SNP were transformed to 0, 1, and 2, respectively. The regression analysis was performed between normalized nitric oxide response of Monocyte-Derived Macrophages culture treated by *Escherichia coli* (n = 58) and numerically coded SNPs. To account for multiple comparison errors, permutation analysis (10,000 iterations) analysis for each SNP and the permutation p-value of 0.0005 were considered as the threshold for each SNPs. Significant SNPs are shown in red.

**Figure 3:**
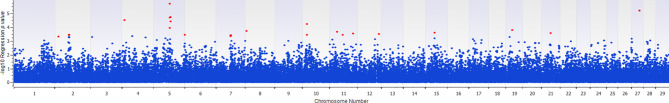
Distribution of -Log10 regression p values from dominant genetic model analysis of SNPs in association with MDM nitric oxide production. In this analysis, the alphabetic codes of aa, ab, and bb for each SNP were transformed to 0, 1, and 1, respectively. The regression analysis was performed between normalized nitric oxide response of Monocyte-Derived Macrophages culture treated by *Escherichia coli* (n = 58) and numerically coded SNPs. To account for multiple comparison errors, permutation analysis (10,000 iterations) analysis for each SNP and the permutation p-value of 0.0005 were considered as the threshold for each SNPs. Significant SNPs are shown in red.

**Figure 4:**
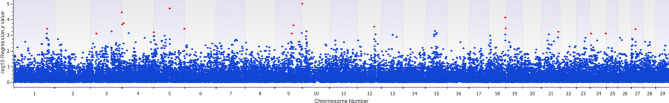
Distribution of -Log10 regression p values from recessive genetic model analysis of SNPs in association with MDM nitric oxide production. In this analysis, the alphabetic codes of aa, ab, and bb for each SNP were transformed to 0, 0, and 1, respectively. The regression analysis was performed between normalized nitric oxide response of Monocyte-Derived Macrophages culture treated by *Escherichia coli* (n = 58) and numerically coded SNPs. To account for multiple comparison errors, permutation analysis (10,000 iterations) analysis for each SNP and the permutation p-value of 0.0005 were considered as the threshold for each SNPs. Significant SNPs are shown in red.

**Figure 5:**
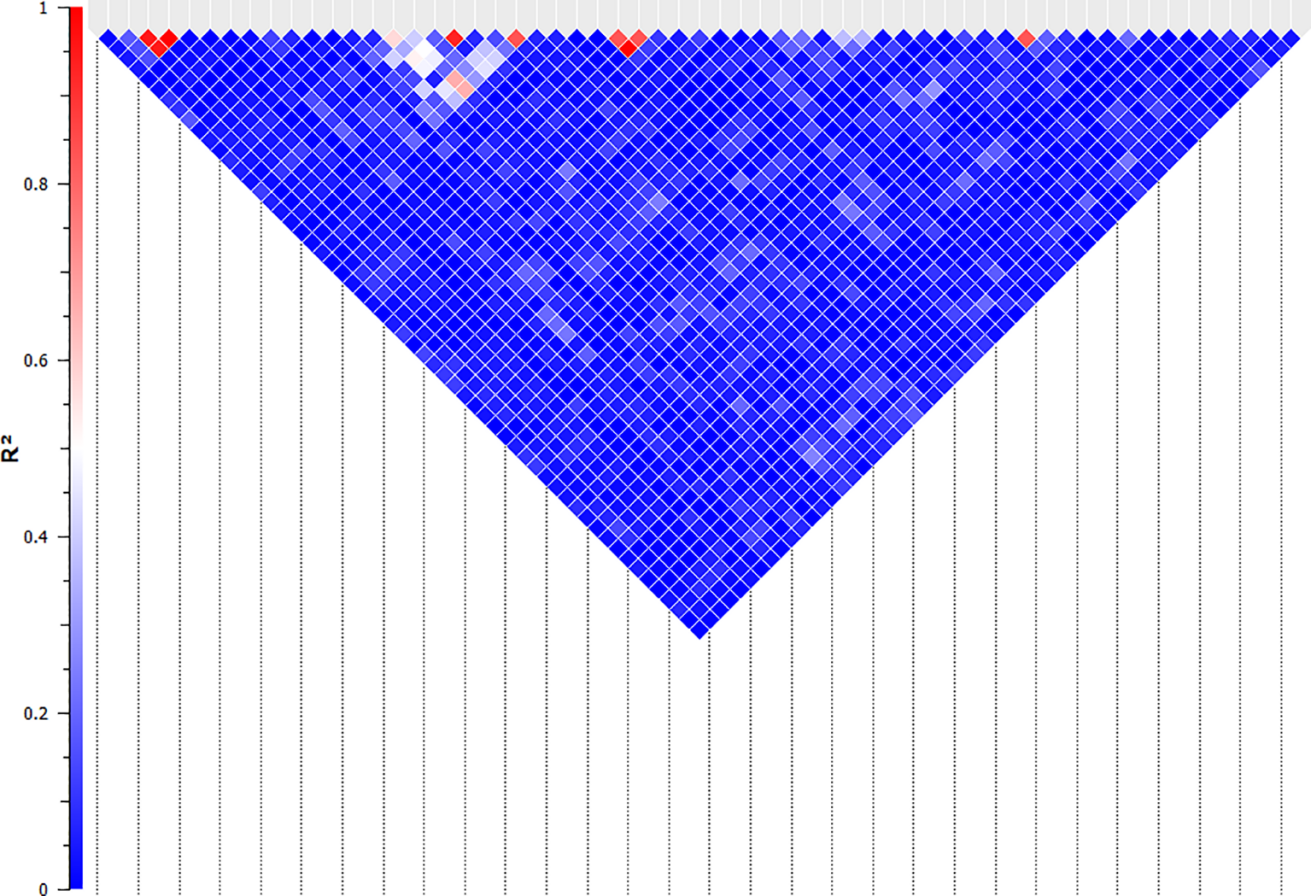
Linkage analysis between the SNPs that are significantly associated with NO^-^ production in bovine monocyte-derived macrophages. The linkage between the significant SNPs was evaluated using the Expectation-Maximization algorithm and evaluated by R-Squared (represented by the colour of the square between two SNPs) in SNP & Variation Suite (ver. 8.8.1, Golden Helix Inc.). The SNPs with R-Squared of greater than 0.5 were considered linked and the second SNPs in each pair were removed for the quantitative analysis.

**Figure 6:**
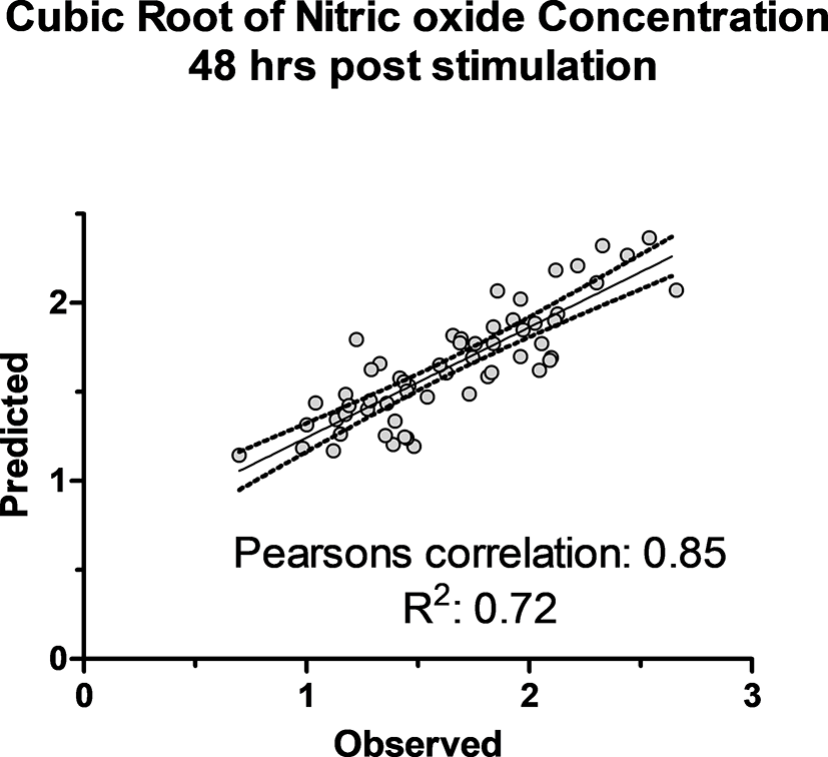
The observed cubic root of nitric oxide concentration versus predicted concentration using 51 SNPs in K-fold genomic prediction algorithm in SNP & Variation Suite (Golden Helix Inc.) using Bayes C(π) method in 50,000 iterations in 6 folds of prediction.

The association of 60 SNPs with the concentration of NO^-^ was found to be statistically significant (permutation value < 0.0005). These SNPs were located on 22 autosomal chromosomes. The pattern of genetic effects for 22, 20 and 18 SNPs followed additive, dominant, and recessive models, respectively (**Figures 2**–**4** and **Supplementary File 1**, **Sheet Table 1**). The linkage analysis showed strong LD among 9 pairs of SNPs (**Figure 5**). The 51 informative SNPs were able to predict the NO^-^ concentration with 85% correlation between the observed and the predicted NO^-^ concentration with an R-Squared of 0.708 for the linear regression between the observed and the predicted NO^-^ concentration (**Figure 6**). This R-Squared was considered as the genomic heritability of the NO^-^ index (45).

**Figure 7:**
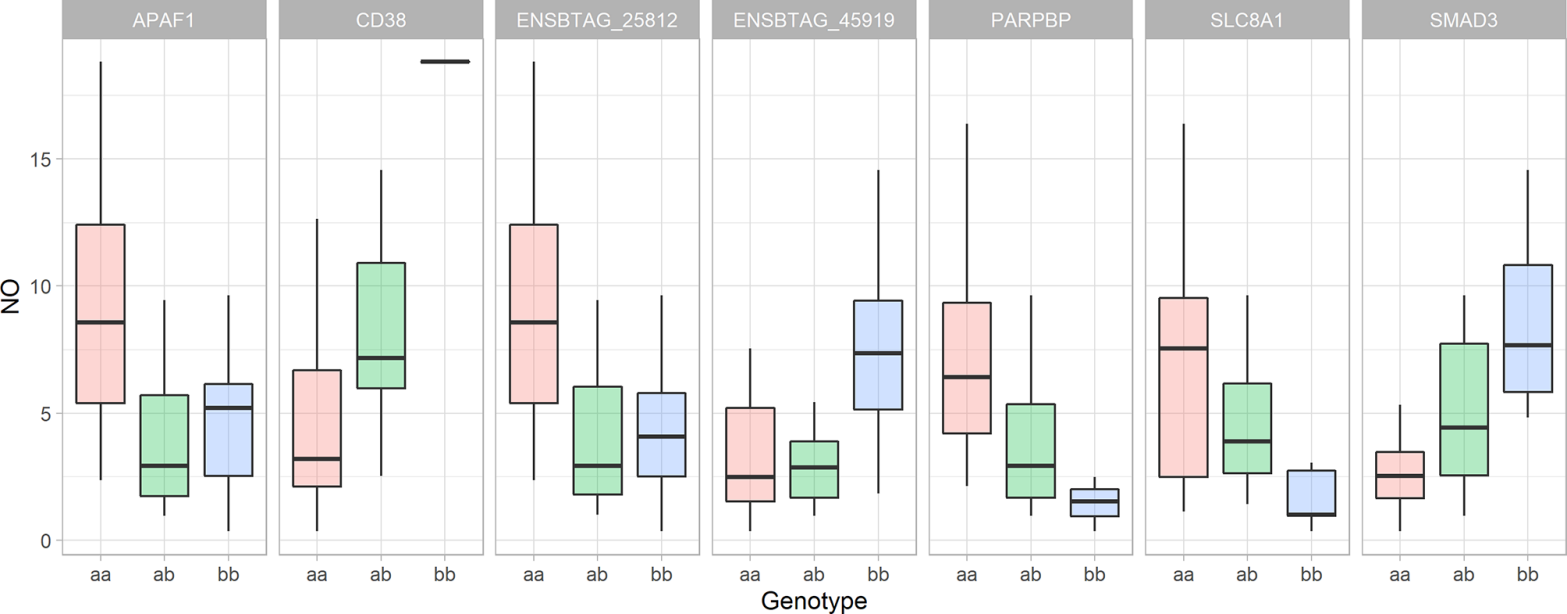
The Whiskers plots of nitric oxide concentration in the culture supernatant of bovine monocyte-derived macrophages 48 hours after exposure to Escherichia coli based on the cow’s genotype for significant SNPs nearby genes with role in response to hypoxia. The y-axis represents the nitric oxide concentration in µM, the animal genotypes are represented along the x-axis. “The lower and upper hinges correspond to the first and third quartiles. The upper whisker extends from the hinge to the largest value no further than 1.5 * IQR from the hinge (where IQR is the inter-quartile range, or distance between the first and third quartiles). The lower whisker extends from the hinge to the smallest value at most 1.5 * IQR of the hinge.” (Summary of statistics for geom_boxplot function in ‘ggplot2’ R package).

By employing the position-dependent strategy, 72 genes, including 14 novel genes, 8 non-coding genes and 2 micro-RNA were identified as positional candidate genes. By employing the comparative genomic approach, 8 coding genes were identified based on the similarity to the human genome. No genes were found in the vicinity of the 8 SNPs at the end of screening for the positional candidate genes (**Supplementary File 1** - **Sheet Table 1**). The top 3 R-Squared for a single SNP were 0.413 (adjacent to *THBS2*), 0.332 (adjacent to *PARPBP*), and 0.306 (adjacent to ENSBTAG00000045919) (**Figure 7**). After removing all non-coding and novel genes and combining genes from all 3 steps of the screening, the final list of positional candidate genes consisted of 58 genes (**Supplementary File 1** - **Sheet Table 1**). In this set of 58 genes, the “vesicle-mediated transport” GO term (GO:0016192) was in common between 8 genes (*ARL1, STXBP6, PIK3C2A, SORL1, YIPF5, MCFD2, STX2*, and *VAMP4*), calcium-related GO terms of “calcium ion binding” (GO:0005509), and “calcium:sodium antiporter activity” (GO:0005432), were in common between 6 genes (*SNED1, THBS2, LC8A1, FGF14, CDH20, PDE1A*, and *LRP1B*) and “response to hypoxia” (GO:0001666) were in common between 4 genes (*APAF1*, *CD38*, *SLC18A1*, and *SMAD3*).

**Figure 8:**
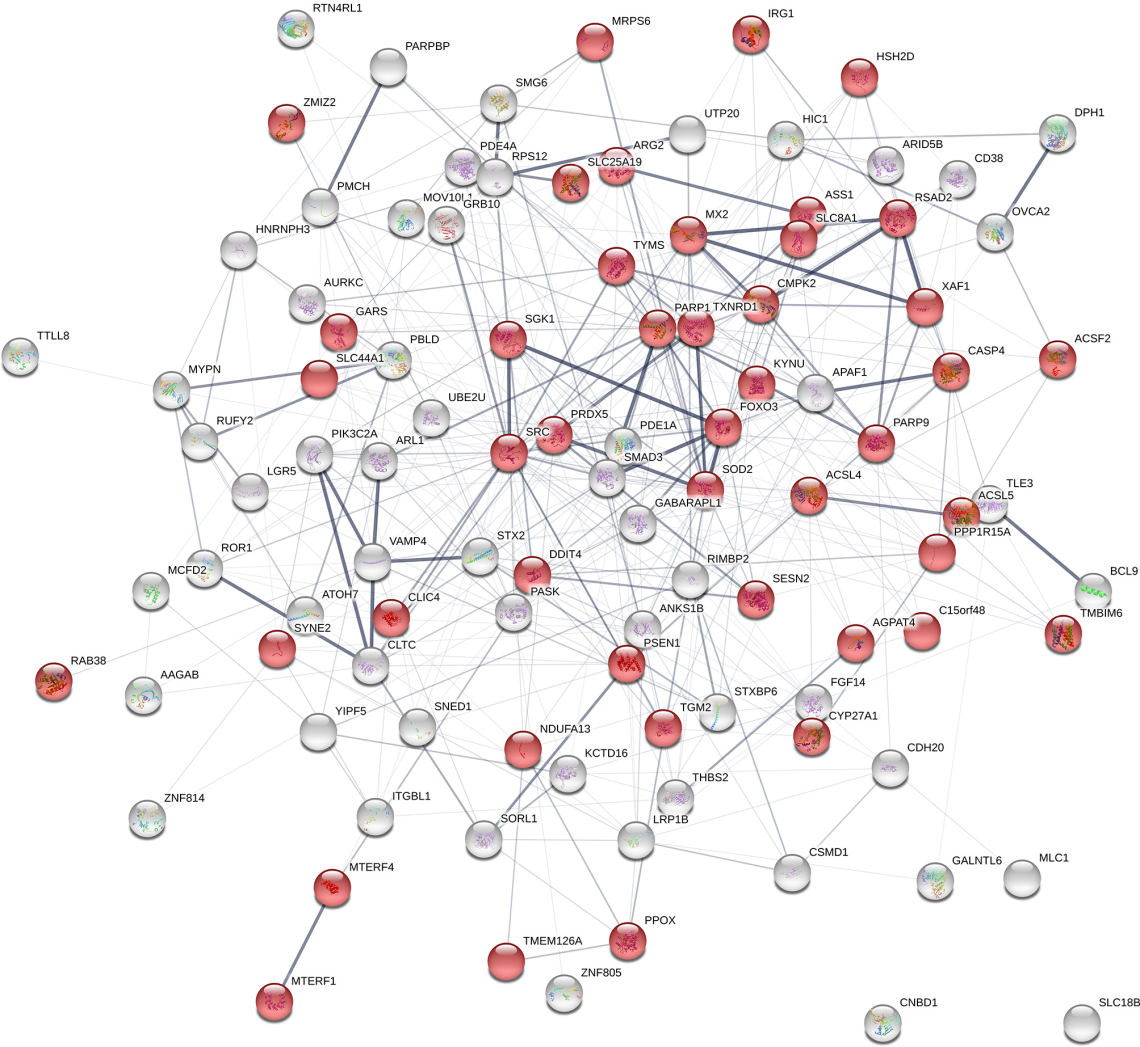
Gene network using the list of positional candidate genes from the current genome-wide association studies and the differentially expressed genes that are annotated with “mitochondrion” The genes that are annotated with “mitochondrion” GO term are shown in red. Number of nodes: 102; Number of edges: 439; Average node degree: 8.61; Average local clustering coefficient: 0.345; Expected number of edges: 354; PPI enrichment p-value: 6.87e-06.

From the previously data set of 839 DE genes between high and low NO^-^ responders (Fold change > 1.5 and FDR p-value < 0.1), 44 genes were found to be annotated with the GO term of “mitochondrion” (GO:0005739) (**Supplementary File 1** - **Sheet Table 3**). The interaction analysis using STRING (v. 11) on the combined list of genes (positional candidate genes and mitochondria-related DE genes = 102 genes) resulted in one network of 100 connected nodes (**Figure 8**). The enrichment *p-value* of this network was 6.87e-6 with the average local clustering coefficient of 0.345. Only two genes, *SLC18B1* and *CNBD1* (both were selected at the first step of screening) were not connected to the network. The functional enrichment analysis on the network showed that “response to oxygen-containing compound” (GO:1901700) and “response to oxidative stress” (GO:0034599) were significantly enriched (FDR ≤ 0.002) (**Supplementary File 1** - **Sheet Table 4**).

## Discussion

Identifying the genetic elements that shape the host response to pathogens has been gaining more attention as the treatment and prognosis of diseases are moving towards precision (veterinary) medicine. Individual differences in response to pathogens or treatments have been observed in outbred populations. However, identifying the genetic mechanisms that control this variation is extremely difficult. More than 8,000 genes in the human genome are known to contribute to immune responses (Ensemble, Genes 97) (46). In addition, the effective population size of the human population is very large (47). The combination of these two factors requires a large sample size to discover genetic control of immunity at the whole-organism level. Reductionist approaches are shown to be a successful alternative to overcome some of the barriers of studies on whole-organisms. Investigating the effect of genetic polymorphism on regulation of subsystems, instead of the whole organism, can overcome the limitation of the complexity of the trait. However, in the reductionist approach, the effect of environment, interactions with other cells and the physiological condition of host are removed which may limit the application of the findings. The genetic control of the sub-systems is much simpler than the whole organism (48). Hence, these studies require smaller sample size and the generated data is free from environmental effects (49). In 2013, the concept of cellular genome-wide association study was proposed by Dennis Ko et al. (50). Since then, this approach has been used to reveal the effect of genetic polymorphism on regulation of host-pathogen interaction (51–53). It has been shown that the genetic associations that are identified in this approach are relevant to resistance to disease based on clinical data (54, 55). Genetic studies using a reductionist approach in livestock can further address the limitation of effective population size in comparison to studies on human. The heritability of NO^-^ production in bovine MDM *in-vitro* was estimated to be 0.78. This heritability is similar to the heritability of the NO^-^ production by mouse peritoneal macrophages (0.8), indicating the majority of variation is described by genetics in this *in-vitro* model (17, 18). Further investigation of the cellular mechanisms shaping the NO-based phenotype showed that one network of transcription factors is controlled by the host genetics. This network was composed of two clusters: hypoxia-related genes including HIF1A and inflammatory-related clusters. The induction of HIF1A under the normoxic condition in macrophages and neutrophils has been reported previously (20, 21). This activation is thought to be hypoxia-independent (20). However, the role of the oxidative burst in the activation of HIF1A was shown by abrogation of the HIF1A pathway when ROS generation was inhibited in macrophages (20, 56). Furthermore, Lue et al. showed that oxidative burst can cause hypoxia at the cellular level and activate HIF1A (57).

In the current study, 60 SNPs were found to be significantly associated with NO- production. Although the sample size of this study seems to be small for a GWAS, sample size is a relative concept. In association studies, it depends on several factors, for instance: the level of genetic diversity in the population, the complexity of the phenotype and its heritability, environmental and physiological effects, and the population structure (58). The effective population size (*N_e_*) of Holstein, based on linkage disequilibrium, has been estimated to be approximately 58 (59). The small *N_e_* indicates a high level of inbreeding, limited genetic diversity and long genetic linkage (59, 60). The phenotype that was analyzed in the current GWAS is a relatively simple trait and highly heritable (h^2^: 0.77-0.99), measured in a fully controlled environment, and shown to be free from physiological effects (17). In addition, samples were collected from one sex and one breed to reduce the possibility of population stratification. Considering these factors, the sample population was sufficient to identify loci that are significantly associated with the trait. A similar design in a study on the human population has successfully identified loci that are associated with cytokine response by using 500 samples (51). It should be noted the effective population size of the aforementioned human population was approximately 150,000 which is 2,500 fold larger than effective population size of Canadian Holsteins (61). When the effective population size is smaller, much lower samples will be adequate to find QTLs (61). This concept in human studies is known as familial GWAS or GWAS on isolated population (62). In cattle, 50 samples and less were successfully used in a genome-wide eQTL mapping analysis and GWAS (63, 64).

The similarity between the genomic heritability and the pedigree-based heritability indicates that the identified loci are the main genetic regulators of macrophage pro-inflammatory responses. The significant interaction network between the genes that are nearby significant SNPs and mitochondria-related genes that were associated with the NO^-^ index, indicates the role of mitochondria in the regulation of the inflammatory profile of MDM. The functional annotation analysis of the network showed that “response to oxygen-containing compound” (GO: 1901700) was the most enriched biological process in the network. “Response to hypoxia” was one of the other enriched terms among 4 positional candidate genes. The SNPs nearby genes that are annotated with the response to hypoxia (*APAF1*, *CD38*, *SMAD3*, and *SLC8A1*) described a notable proportion of the observed variation (33.1%, 26.4%, 18.5%, and 16.9%, respectively). In addition, the SNPs that are nearby two novel genes, ENSBTAG00000025812 (an orthologue of CD38) and ENSBTAG00000045919 (has a role in oxidative stress-induced sensing, Reactome pathway: R-BTA-2559580), explained 32.4% and 30.6% of the observed variation, respectively. The SNP that is nearby PARPBP described 24.9% of the observed variation. Although this gene does not have a direct role in hypoxia, the product of this gene binds to PARP1 and enhance its function (65). PARP1 is an ADP-ribose polymerase which can be found in mitochondria with a role in the cellular response to oxidative stress (GO:0034599). Our previous study has shown that *PARP1* is significantly downregulated in NO- high responder macrophages. The PARP1 gene has a positive role in mitochondrial depolarization (GO:0051901). Depolarization of the mitochondrial membrane is a mechanism that results in sensitivity to hypoxic condition and inability to cope with hypoxic conditions (66). By only including SNPs nearby genes that are associated with response to hypoxia (*APAF1*, *CD38*, *SMAD3*, *SLC8A1, PARPBP*, ENSBTAG00000025812, and ENSBTAG00000045919) in the general linear model (to include additive and non-additive genetic effects), 72.9% of the variation in NO^-^ response was described by these 7 SNPs. Therefore, the difference between the broad-sense heritability (0.80) of macrophage NO^-^ production and the R-Squared of the aforementioned model indicates that approximately 7% of the genetic regulation of macrophage response is controlled by the other 53 SNPs, and probably other loci that were not detected in this study. This large proportion of variation that is described by these 7 SNPs shows that response to hypoxia, probably *via* mitochondria, is the major genetic mechanism that controls the MDMs functional characteristics.

The results of our previous study showed that HIF1A was one of only 4 TFs found to be activated at both 3- and 18-hours post-exposure to *E. coli*. In addition, the cluster containing the *HIF1A* was expanded at 18 hours in comparison to 3 hours. The synergic interaction between *HIF1A* and NFkB in macrophages can lead to increased production of NO^-^ (67). We have previously shown a strong positive correlation between NO^-^ production and phagocytosis (17). It probably indicates that the pathway from phagocytosis to oxidative burst, hypoxia, HIF1A activation, pro-inflammatory response in macrophages is a dose-dependent pathway.

The role of other candidate genes (i.e. PASK), biological process (i.e. “vesicle-mediated transport”), molecular function (i.e. “calcium ion binding”, and “calcium:sodium antiporter activity”) that were found in the current study should also be emphasized. *PASK* codes a protein that is a nutrient-sensing molecule with a role in regulating cellular energy balance *via* regulating lipid and glucose metabolism, mitochondrial respiration and phosphorylation (68, 69). The role of Ca^+^-depended exocytosis in macrophages is an example of the interaction between the “vesicle-mediated transport” and Ca ions that regulates phagocytosis which is the primary triggers of the oxidative burst-induced hypoxia (57, 70).

In conclusion, the findings of the current study align with the previously published mechanisms in mice and humans regarding the role of mitochondria and response to hypoxia controlled by HIF1A of macrophages, thereby broadening the importance of this mechanism across species. Furthermore, these results show the effect of genetic polymorphism on regulation of response to oxidative burst-induced hypoxia is a major mechanism that controls macrophage pro-inflammatory responses such as NO^-^ production. It should be mentioned that, although this genetic predisposition is reported in MDM in the current study, the genes are shared among all leukocytes of the host. Hence, in an individual who carries unfavourable alleles, other cells, such as neutrophils and monocytes, that are capable of oxidative burst will likely also share an impaired response (71, 72). Also, if the hypoxic condition is caused by other factors such as inflammation or solid tumours, then all the cells of the immune system in a hypoxic tissue (such as lymphocytes) may not be able to maintain their function and cope with the hypoxic condition (73). Therefore, the final consequence of genetic sensitivity to hypoxia will likely have a broader effect on the outcome of infection than just the effects that are directly or indirectly linked to macrophages. It should also worth noting that the results of the current study may not fully represent the function of macrophages in the host. Macrophages in the host are transformed from monocytes in a dynamic environment and work in collaboration with other cells of the immune system. These interactions are missing in the model that we used. Moreover, the phase of quantitative trait loci is different between breeds of cattle, and the results of the current study should be validated on other populations.

## Data Availability Statement

The datasets presented in this study can be found in online repositories. The names of the repository/repositories and accession number(s) can be found in the article/**Supplementary Material**.

## Ethics Statement

The animal study was reviewed and approved by The animal care committee of the University of Guelph.

## Author Contributions

ME and BM conceptualized the study and designed the experiments. ME and ST ran the experiments. ME and MS ran the statistical procedures. ME drafted the manuscript and ST, MS, and BM critically reviewed the manuscript. BM spearheaded the project as the principal investigator. All authors contributed to the article and approved the submitted version.

## Funding

This research was funded by grants to BM from the Natural Sciences and Engineering Research Council (NSERC). This paper is also a contribution to the Food from Thought research program supported by the Canada First Research Excellence Fund. The funders did not have any role in the design, collection, analysis, and interpretation of data and in writing the manuscript. ME was supported by the Natural Sciences and Engineering Research Council *via* scholarship.

## Conflict of Interest

MS was employed by Select Sires Inc. The commercial use of the phenotyping method of Monocyte-Derived Macrophages that was described in the manuscript is protected by the Patent Cooperation Treaty (PCT/CA2020/050997) and pending US patents (App. No. 62/878,377 and 62/941,927). ME is the founder of Biomics Analytica Inc. and BM is the CEO of Immunogenetic Inc. These companies are commercializing the SNPs that were described in the manuscript.

The remaining authors declare that the research was conducted in the absence of any commercial or financial relationships that could be construed as a potential conflict of interest.

## Supplementary Material

Supplementary Table 1 The list of SNPs that are significantly associated with the concentration of nitric oxide in the supernatant of Monocyte-Derived Macrophages culture treated by *Escherichia coli* after 48 hours exposure. The genes that were selected in the second step of screening (expanding the window around the significant SNPs) are shown by “+” sign. The genes that are selected based on the 3^rd^ step of screening (similarity to the human genome) are shown by “*” sign.

Supplementary Table 2 The list of differentially expressed genes from the previous transcriptomic study that are annotated with “mitochondrion” GO term.

Supplementary Table 3 The list of biological process GO terms that were enriched in the interaction network.

Supplementary Table 4 The list of biological process terms over-represented in the network of protein-protein interaction analysis (shown in **Figure 8**) of differentially expressed genes (GSE136722) and the positional candidate genes from genome-wide association analysis of the current study.

## References

[1] Lavin Y Mortha A Rahman A Merad M. Regulation of Macrophage Development and Function in Peripheral Tissues. Nat Rev Immunol (2015) 15:731–44. 10.1038/nri3920 PMC4706379 26603899

[2] Soehnlein O Lindbom L. Phagocyte Partnership During the Onset and Resolution of Inflammation. Nat Rev Immunol (2010) 10:427–39. 10.1038/nri2779 20498669

[3] Liew FY Parish CR. Regulation of the Immune Response by Antibody. 3. The Effect of Macrophage Cytophilic Antibody on Humoral and Cell-Mediated Immunity. Cell Immunol (1972) 5:520–35. 10.1016/0008-8749(72)90102-5 4539284

[4] Bain CC Bravo-Blas A Scott CL Perdiguero EG Geissmann F Henri S. Constant Replenishment From Circulating Monocytes Maintains the Macrophage Pool in the Intestine of Adult Mice. Nat Immunol (2014) 15:929–37. 10.1038/ni.2967 PMC4169290 25151491

[5] Ginhoux F Schultze JL Murray PJ Ochando J Biswas SK. New Insights Into the Multidimensional Concept of Macrophage Ontogeny, Activation and Function. Nat Immunol (2016) 17:34–40. 10.1038/ni.3324 26681460

[6] Italiani P Boraschi D. Development and Functional Differentiation of Tissue-Resident Versus Monocyte-Derived Macrophages in Inflammatory Reactions. In: Results and Problems in Cell Differentiation (2017). p. 23–43. 10.1007/978-3-319-54090-0_2 28455704

[7] Gerrick KY Gerrick ER Gupta A Wheelan SJ Yegnasubramanian S Jaffee EM. Transcriptional Profiling Identifies Novel Regulators of Macrophage Polarization. PloS One (2018) 13:e0208602. 10.1371/journal.pone.0208602 30532146 PMC6286176

[8] Wang N Liang H Zen K. Molecular Mechanisms That Influence the Macrophage M1 – M2 Polarization Balance. Front. Immunol. (2014) 5:1–9. 10.3389/fimmu.2014.00614 25506346 PMC4246889

[9] Lawrence T Natoli G. Transcriptional Regulation of Macrophage Polarization: Enabling Diversity With Identity. Nat Rev Immunol (2011) 11:750–61. 10.1038/nri3088 22025054

[10] Gosselin D Glass CK. Epigenomics of Macrophages. Immunol Rev (2014) 262:96–112. 10.1111/imr.12213 25319330 PMC4203424

[11] Glass CK Natoli G. Molecular Control of Activation and Priming in Macrophages. Nat Immunol (2015) 17:26–33. 10.1038/ni.3306 PMC4795476 26681459

[12] Saeed S Quintin J Kerstens HHD Rao NA Aghajanirefah A Matarese F. Epigenetic Programming of Monocyte-to-Macrophage Differentiation and Trained Innate Immunity. Science (2014) 345:1251086–1251086. 10.1126/science.1251086 25258085 PMC4242194

[13] Langlais D Barreiro LB Gros P. The Macrophage IRF8/IRF1 Regulome Is Required for Protection Against Infections and Is Associated With Chronic Inflammation. J Exp Med (2016) 213:585–603. 10.1084/jem.20151764 27001747 PMC4821649

[14] Romanoski CE Link VM Heinz S Glass CK. Exploiting Genomics and Natural Genetic Variation to Decode Macrophage Enhancers. Trends Immunol (2015) 36:507–18. 10.1016/j.it.2015.07.006 PMC4548828 26298065

[15] Glass CK. Genetic and Genomic Approaches to Understanding Macrophage Identity and FunctionATVB Named Lecture Reviews—Insight Into Author. Arterioscler Thromb Vasc Biol (2015) 35:755–62. 10.1161/ATVBAHA.114.304051 PMC4376616 25745059

[16] Netea MG Joosten LA. Master and Commander: Epigenetic Regulation of Macrophages. Cell Res (2016) 26:145–6. 10.1038/cr.2016.5 PMC4746612 26768770

[17] Emam M Tabatabaei S Sargolzaei M Sharif S Schenkel F Mallard B. The Effect of Host Genetics on *In Vitro* Performance of Bovine Monocyte-Derived Macrophages. J Dairy Sci (2019) 102:9107–16. 10.3168/JDS.2018-15960 31400895

[18] Zídek Z Franková D Boubelík M. Genetic Variation in *In-Vitro* Cytokine-Induced Production of Nitric Oxide by Murine Peritoneal Macrophages. Pharmacogenetics (2000) 10:493–501. 10.1097/00008571-200008000-00002 10975603

[19] Emam M Cánovas A Islas-Trejo AD Fonseca PAS Medrano JF Mallard B. Transcriptomic Profiles of Monocyte-Derived Macrophages in Response to Escherichia Coli Is Associated With the Host Genetics. Sci Rep (2020) 10. 10.1038/s41598-019-57089-0 PMC6959288 31937813

[20] Nishi K Oda T Takabuchi S Oda S Fukuda K Adachi T. LPS Induces Hypoxia-Inducible Factor 1 Activation in Macrophage-Differentiated Cells in a Reactive Oxygen Species–Dependent Manner. Antioxid Redox Signal (2008) 10:983–96. 10.1089/ars.2007.1825 18199003

[21] Wang T Liu H Lian G Zhang S-Y Wang X Jiang C. Hif1α-Induced Glycolysis Metabolism Is Essential to the Activation of Inflammatory Macrophages. Mediators Inflammation (2017) 2017:9029327. 10.1155/2017/9029327 PMC5745720 29386753

[22] Tur J Vico T Lloberas J Zorzano A Celada A. Macrophages and Mitochondria. In: Advances in Immunology (2017). p. 1–36. 10.1016/bs.ai.2016.12.001 28215277

[23] Mills EL Kelly B O’Neill LAJ. Mitochondria Are the Powerhouses of Immunity. Nat Immunol (2017) 18:488–98. 10.1038/ni.3704 28418387

[24] Fuhrmann DC Brüne B. Mitochondrial Composition and Function Under the Control of Hypoxia. Redox Biol (2017) 12:208–15. 10.1016/j.redox.2017.02.012 PMC5333533 28259101

[25] Liu P-S Ho P-C. Mitochondria: A Master Regulator in Macrophage and T Cell Immunity. Mitochondrion (2018) 41:45–50. 10.1016/j.mito.2017.11.002 29146487

[26] Meyer A Laverny G Bernardi L Charles AL Alsaleh G Pottecher J. Mitochondria: An Organelle of Bacterial Origin Controlling Inflammation. Front Immunol (2018) 9:536. 10.3389/fimmu.2018.00536 29725325 PMC5916961

[27] Dölle C Rack JGM Ziegler M. NAD and ADP-Ribose Metabolism in Mitochondria. FEBS J (2013) 280:3530–41. 10.1111/febs.12304 23617329

[28] Li W Sauve AA. NAD^+^ Content and Its Role in Mitochondria. Methods Mol Biol (2015) 1241:39–48. 10.1007/978-1-4939-1875-1_4 25308486

[29] VanLinden MR Dölle C Pettersen IKN Kulikova VA Niere M Agrimi G. Subcellular Distribution of NAD+ Between Cytosol and Mitochondria Determines the Metabolic Profile of Human Cells. J Biol Chem (2015) 290:27644–59. 10.1074/jbc.M115.654129 PMC4646015 26432643

[30] Njoroge JM Mitchell LB Centola M Kastner D Raffeld M Miller JL. Characterization of Viable Autofluorescent Macrophages Among Cultured Peripheral Blood Mononuclear Cells. Cytometry (2001) 44:38–44. 10.1002/1097-0320(20010501)44:1<38::AID-CYTO1080>3.0.CO;2-T 11309807

[31] Mitchell AJ Pradel LC Chasson L Van Rooijen N Grau GE Hunt NH. Technical Advance: Autofluorescence as a Tool for Myeloid Cell Analysis. J Leukoc Biol (2010) 88:597–603. 10.1189/jlb.0310184 20534703

[32] Fuentes-Duculan J Suárez-Fariñas M Zaba LC Nograles KE Pierson KC Mitsui H. A Subpopulation of CD163-Positive Macrophages Is Classically Activated in Psoriasis. J Invest Dermatol (2010) 130:2412–22. 10.1038/jid.2010.165 PMC2939947 20555352

[33] Wiggans GR Sonstegard TS VanRaden PM Matukumalli LK Schnabel RD Taylor JF. Selection of Single-Nucleotide Polymorphisms and Quality of Genotypes Used in Genomic Evaluation of Dairy Cattle in the United States and Canada. J Dairy Sci (2009) 92:3431–6. 10.3168/jds.2008-1758 19528621

[34] Sargolzaei M Chesnais JP Schenkel FS. A New Approach for Efficient Genotype Imputation Using Information From Relatives. BMC Genomics (2014) 15:478. 10.1186/1471-2164-15-478 24935670 PMC4076979

[35] Krishnamoorthy K Mathew T Mukherjee S. Normal-Based Methods for a Gamma Distribution. Technometrics (2008) 50:69–78. 10.1198/004017007000000353

[36] de Klerk B Emam M Thompson-Crispi KA Sargolzaei M van der Poel JJ Mallard BA. A Genome-Wide Association Study for Natural Antibodies Measured in Blood of Canadian Holstein Cows. BMC Genomics (2018) 19:694. 10.1186/s12864-018-5062-6 30241501 PMC6150957

[37] Loker S Miglior F Bohmanova J Jamrozik J Schaeffer LR. Phenotypic Analysis of Pregnancy Effect on Milk, Fat, and Protein Yields of Canadian Ayrshire, Jersey, Brown Swiss, and Guernsey Breeds. J Dairy Sci (2009) 92:1300–12. 10.3168/JDS.2008-1425 19233823

[38] Ibeagha-Awemu EM Peters SO Akwanji KA Imumorin IG Zhao X. High Density Genome Wide Genotyping-by-Sequencing and Association Identifies Common and Low Frequency SNPs, and Novel Candidate Genes Influencing Cow Milk Traits. Sci Rep (2016) 6:31109. 10.1038/SREP31109 27506634 PMC4979022

[39] Clarke GM Anderson CA Pettersson FH Cardon LR Morris AP Zondervan KT. Basic Statistical Analysis in Genetic Case-Control Studies. Nat Protoc (2011) 6:121–33. 10.1038/nprot.2010.182 PMC3154648 21293453

[40] Excoffier L Slatkin M. Maximum-Likelihood Estimation of Molecular Haplotype Frequencies in a Diploid Population. Mol Biol Evol (1995) 12:921–7. 10.1093/oxfordjournals.molbev.a040269 7476138

[41] Habier D Fernando RL Kizilkaya K Garrick DJ. Extension of the Bayesian Alphabet for Genomic Selection. BMC Bioinf (2011) 12:186. 10.1186/1471-2105-12-186 PMC3144464 21605355

[42] Zhu M Zhao S. Candidate Gene Identification Approach: Progress and Challenges. Int J Biol Sci (2007) 3:420–7. 10.7150/ijbs.3.420 PMC2043166 17998950

[43] Durinck S Spellman PT Birney E Huber W. Mapping Identifiers for the Integration of Genomic Datasets With the R/Bioconductor Package Biomart. Nat Protoc (2009) 4:1184–91. 10.1038/nprot.2009.97 PMC3159387 19617889

[44] Szklarczyk D Gable AL Lyon D Junge A Wyder S Huerta-Cepas J. String v11: Protein–Protein Association Networks With Increased Coverage, Supporting Functional Discovery in Genome-Wide Experimental Datasets. Nucleic Acids Res (2019) 47:D607–13. 10.1093/nar/gky1131 PMC6323986 30476243

[45] Kim H Grueneberg A Vazquez AI Hsu S de Los Campos G. Will Big Data Close the Missing Heritability Gap? Genetics (2017) 207:1135–45. 10.1534/genetics.117.300271 PMC5676235 28893854

[46] Hunt SE McLaren W Gil L Thormann A Schuilenburg H Sheppard D. Ensembl Variation Resources. Database (Oxford) (2018) 2018:bay119. 10.1093/database/bay119 PMC6310513 30576484

[47] PARK L. Effective Population Size of Current Human Population. Genet Res (Camb) (2011) 93:105–14. 10.1017/S0016672310000558 21450133

[48] Kemper KE Littlejohn MD Lopdell T Hayes BJ Bennett LE Williams RP. Leveraging Genetically Simple Traits to Identify Small-Effect Variants for Complex Phenotypes. BMC Genomics (2016) 17:858. 10.1186/s12864-016-3175-3 27809761 PMC5094043

[49] Miller S Chaudhary A. A Cellular Gwas Approach to Define Human Variation in Cellular Pathways Important to Inflammation. Pathogens (2016) 5:39. 10.3390/pathogens5020039 PMC4931390 27128945

[50] Ko DC Urban TJ Pozzoli U Ferrer-Admetlla A Pattini L. Understanding Human Variation in Infectious Disease Susceptibility Through Clinical and Cellular Gwas. PloS Pathog (2013) 9:e1003424. 10.1371/journal.ppat.1003424 23935492 PMC3731241

[51] Li Y Oosting M Smeekens SP Jaeger M Aguirre-Gamboa R Le KTT. A Functional Genomics Approach to Understand Variation in Cytokine Production in Humans. Cell (2016) 167:1099–110.e14. 10.1016/j.cell.2016.10.017 27814507

[52] Li Y Oosting M Deelen P Ricaño-Ponce I Smeekens S Jaeger M. Inter-Individual Variability and Genetic Influences on Cytokine Responses to Bacteria and Fungi. Nat Med (2016) 22:952–60. 10.1038/nm.4139 PMC5084084 27376574

[53] ter Horst R Jaeger M Smeekens SP Oosting M Swertz MA Li Y. Host and Environmental Factors Influencing Individual Human Cytokine Responses. Cell (2016) 167:1111–24.e13. 10.1016/j.cell.2016.10.018 27814508 PMC5787854

[54] Wang L Pittman KJ Barker JR Salinas RE Stanaway IB Williams GD. An Atlas of Genetic Variation Linking Pathogen-Induced Cellular Traits to Human Disease. Cell Host Microbe (2018) 24:308–23.e6. 10.1016/j.chom.2018.07.007 30092202 PMC6093297

[55] Alvarez MI Ko DC. Reply to Gilchrist et al.: Possible Roles for VAC14 in Multiple Infectious Diseases. Proc Natl Acad Sci USA (2018) 115:E3604–5. 10.1073/pnas.1803533115 PMC5910875 29588421

[56] Moon EJ Sonveaux P Porporato PE Danhier P Gallez B Batinic-Haberle I. NADPH Oxidase-Mediated Reactive Oxygen Species Production Activates Hypoxia-Inducible Factor-1 (HIF-1) Via the ERK Pathway After Hyperthermia Treatment. Proc Natl Acad Sci USA (2010) 107:20477–82. 10.1073/pnas.1006646107 PMC2996638 21059928

[57] Luo B Wang J Liu Z Shen Z Shi R Liu Y-Q. Phagocyte Respiratory Burst Activates Macrophage Erythropoietin Signalling to Promote Acute Inflammation Resolution. Nat Commun (2016) 7:12177. 10.1038/ncomms12177 27397585 PMC4942576

[58] Ball RD. Designing a GWAS: Power, Sample Size, and Data Structure (2013). p. 37–98. 10.1007/978-1-62703-447-0_3 23756887

[59] Makanjuola BO Miglior F Abdalla EA Maltecca C Schenkel FS Baes CF. Effect of Genomic Selection on Rate of Inbreeding and Coancestry and Effective Population Size of Holstein and Jersey Cattle Populations. J Dairy Sci (2020) 103:5183–99. 10.3168/jds.2019-18013 32278553

[60] McKay SD Schnabel RD Murdoch BM Matukumalli LK Aerts J Coppieters W. Whole Genome Linkage Disequilibrium Maps in Cattle. BMC Genet (2007) 8:74. 10.1186/1471-2156-8-74 17961247 PMC2174945

[61] Somers M Olde Loohuis LM Aukes MF Pasaniuc B De Visser KCL Kahn RS. A Genetic Population Isolate in the Netherlands Showing Extensive Haplotype Sharing and Long Regions of Homozygosity. Genes (Basel) (2017) 8:133. 10.3390/genes8050133 PMC5448007 28471380

[62] Kristiansson K Naukkarinen J Peltonen L. Isolated Populations and Complex Disease Gene Identification. Genome Biol (2008) 9:109. 10.1186/gb-2008-9-8-109 18771588 PMC2575505

[63] Higgins MG Fitzsimons C McClure MC McKenna C Conroy S Kenny DA. GWAS and eQTL Analysis Identifies a SNP Associated With Both Residual Feed Intake and GFRA2 Expression in Beef Cattle. Sci Rep (2018) 8:14301. 10.1038/s41598-018-32374-6 30250203 PMC6155370

[64] Ariel O Brouard J-S Marete A Miglior F Ibeagha-Awemu E Bissonnette N. Genome-Wide Association Analysis Identified Both RNA-seq and DNA Variants Associated to Paratuberculosis in Canadian Holstein Cattle ‘*In Vitro*’ Experimentally Infected Macrophages. BMC Genomics (2021) 22:162. 10.1186/s12864-021-07487-4 33678157 PMC7938594

[65] Piao L Nakagawa H Ueda K Chung S Kashiwaya K Eguchi H. C12orf48, Termed PARP-1 Binding Protein, Enhances Poly(ADP-Ribose) Polymerase-1 (PARP-1) Activity and Protects Pancreatic Cancer Cells From DNA Damage. Genes Chromosom Cancer (2011) 50:13–24. 10.1002/gcc.20828 20931645

[66] Turcotte ML Parliament M Franko A Allalunis-Turner J. Variation in Mitochondrial Function in Hypoxia-Sensitive and Hypoxia-Tolerant Human Glioma Cells. Br J Cancer (2002) 86:619–24. 10.1038/sj.bjc.6600087 PMC2375290 11870546

[67] Wiese M Gerlach RG Popp I Matuszak J Mahapatro M Castiglione K. Hypoxia-Mediated Impairment of the Mitochondrial Respiratory Chain Inhibits the Bactericidal Activity of Macrophages. Infect Immun (2012) 80:1455–66. 10.1128/IAI.05972-11 PMC3318416 22252868

[68] Zhang D Zhang J Wang Y Liu Y Liu G Li X. Per-Arnt-Sim Kinase (PASK): An Emerging Regulator of Mammalian Glucose and Lipid Metabolism. Nutrients (2015) 7:7437–50. 10.3390/nu7095347 PMC4586542 26371032

[69] Hao H-X Cardon CM Swiatek W Cooksey RC Smith TL Wilde J. PAS Kinase Is Required for Normal Cellular Energy Balance. Proc Natl Acad Sci USA (2007) 104:15466–71. 10.1073/pnas.0705407104 PMC2000499 17878307

[70] Vashi N Andrabi SBA Ghanwat S Suar M Kumar D. Ca2+-Dependent Focal Exocytosis of Golgi-Derived Vesicles Helps Phagocytic Uptake in Macrophages. J Biol Chem (2017) 292:5144–65. 10.1074/jbc.M116.743047 PMC5392664 28174296

[71] Seres T Knickelbein RG Warshaw JB Johnston RB. The Phagocytosis-Associated Respiratory Burst in Human Monocytes Is Associated With Increased Uptake of Glutathione. J Immunol (2000) 165:3333–40. 10.4049/jimmunol.165.6.3333 10975851

[72] Chen Y Junger WG. Measurement of Oxidative Burst in Neutrophils. Methods Mol Biol (2012) 844:115–24. 10.1007/978-1-61779-527-5_8 PMC4214271 22262438

[73] Menk AV Scharping NE Moreci RS Zeng X Guy C Salvatore S. Early TCR Signaling Induces Rapid Aerobic Glycolysis Enabling Distinct Acute T Cell Effector Functions. Cell Rep (2018) 22:1509–21. 10.1016/j.celrep.2018.01.040 PMC5973810 29425506

